# Flubendazole reduces emerging *Dracunculus medinensis* in dogs in Chad: Results from a two-year study

**DOI:** 10.1371/journal.pntd.0014763

**Published:** 2026-09-25

**Authors:** Amy C. Dupper, Charles D. Mackenzie, Timothy G. Geary, Deborah L. Elder, Wided Najahi-Missaoui, Christopher A. Cleveland, Ellen K. Haynes, Michael J. Yabsley, Guilherme Gomes Verocai, Macon Overcast, John A. Bryan II, Fernando Torres-Velez, Métinou Koumétio Sidouin, Bongo Nare Richard Ngandolo, Philip Tchindebet Oaukou, Adam J. Weiss, Rebecca Garabed

**Affiliations:** 1 Division of Epidemiology, College of Public Health, The Ohio State University, Columbus, Ohio, United States of America; 2 Institute of Parasitology, McGill University, Ste-Anne-de-Bellevue, Québec, Canada; 3 School of Biological Sciences, Queen’s University-Belfast, Northern Ireland, United Kingdom; 4 Department of Pharmaceutical and Biomedical Sciences, University of Georgia, Athens, Georgia, United States of America; 5 Southeastern Cooperative Wildlife Diseases Study, University of Georgia College of Veterinary Medicine, Athens, Georgia, United States of America; 6 Center for Ecology of Infectious Diseases, University of Georgia, Athens, Georgia, United States of America; 7 Warnell School of Forestry and Natural Resources, University of Georgia, Athens, Georgia, United States of America; 8 Department of Veterinary Pathology, College of Veterinary Medicine and Biological Sciences, Texas A&M University, College Station, Texas, United States of America; 9 Department of Veterinary Preventive Medicine, College of Veterinary Medicine, The Ohio State University, Columbus, Ohio, United States of America; 10 Zachery Consulting LLC, Ila, Georgia, United States of America; 11 The Carter Center, Atlanta, Georgia, United States of America; 12 The Carter Center, N’Djamena, Chad; 13 Institut de Recherche en Élevage pour le Développement, Afrique One Aspire, N’Djamena, Chad; 14 Programme National d’Éradication du Ver de Guinée, Ministry of Health, N’Djamena, Chad; The University of Melbourne, AUSTRALIA

## Abstract

Since being targeted for eradication in the 1980s, global human cases of dracunculiasis (Guinea worm disease; GWD) have been reduced by over 99.9% through interventions aimed at interrupting the life cycle of the causative parasite, *Dracunculus medinensis*. However, the burden of GWD in dogs is higher than in humans and other monitored mammalian host species, threatening eradication of GWD. Expanding on a previous clinical trial of flubendazole (FLBZ) in dogs in Chad, we conducted a cluster-randomized controlled trial in which half of enrolled villages (N = 29) were assigned to receive FLBZ and the other half (N = 27) to receive no treatment. An annual difference-in-differences analysis revealed that treated villages had 59.3% fewer predicted emerging *D. medinensis* adult females (95% CI: 53.8% fewer to 69.2% fewer) per village per year during the second year of the study compared to control villages. Analyses examining effects of FLBZ monthly, at the individual dog level, and through histopathology of *D. medinensis* female reproductive morphology, all support the conclusion that FLBZ inhibits embryogenesis and larval development within the uterus of gravid female worms. Together these data are consistent with a hypothesis that although *D. medinensis* emerged from individually treated dogs, the L1s released from dogs treated with FLBZ exhibited reduced viability, thus reducing the risk of infection in the next mammalian host. This trial builds the body of knowledge about FLBZ’s impacts on *D. medinensis* in the canine host and provides compelling evidence on the effectiveness of FLBZ in reducing the number of emerging *D. medinensis* in dogs, which can contribute to the eradication of this parasite. However, additional questions remain regarding the formulation, which need to be addressed in future studies before being deployed at scale.

## Author summary

Annual cases of Guinea worm disease (GWD) in humans have been greatly reduced globally through public health interventions such as improving access to clean water, expanding surveillance, treating bodies of water suspected to contain GW larvae with temephos, and educating local populations on reducing their risk of GWD. Dog GW infections remain high compared to human cases, impeding the goal of eradication. We conducted a cluster-randomized clinical trial of flubendazole (FLBZ) in Chadian dogs from October 2021 through June 2023 during which Chad had the highest number of dogs with reported GW infections compared to other endemic countries. All dogs in villages were randomly assigned to receive FLBZ or no treatment. The main annual analysis predicted 59.3% fewer emerging adult female *D. medinensis* (95% CI: 53.8% fewer to 69.2% fewer) per village only during the second year of the study in treated villages compared to control villages. Analyses looking at monthly village-level outcomes, individual dog outcomes, and comparing the histopathology of female worm reproductive morphology all support the hypothesis that FLBZ in this dose and formulation inhibits the viability of 1st-stage larvae that are expelled from gravid female worms such that the risk of infection is decreased in the next mammalian host. This study provides evidence for flubendazole as a potential therapeutic intervention for Guinea worm disease among dogs, though more research is needed regarding the formulation of the drug in both laboratory and field settings. Future studies would provide additional evidence necessary to inform decisions regarding when and how the drug could be deployed at scale.

## Introduction

Dracunculiasis or Guinea worm disease (GWD) is one of the oldest recorded parasitic diseases [1], but the causative agent, *Dracunculus medinensis*, was not described until 1758 by Carolus Linnaeus [2,3]. Infection with *D. medinensis* classically requires the mammalian definitive host to drink water containing copepods, the intermediate host, infected with third-stage larvae (L3) of *D. medinensis* or alternatively ingest an aquatic animal (paratenic or transport host) infected with L3 larvae [4–10]**.** L3s are released into the host’s stomach and migrate out of the intestine to the abdominal wall and thorax where *D. medinensis* larvae develop to the adult stage, sexually mature and reproduce [4,7,11]. The average time from ingestion to emergence of gravid females is approximately 12 months [4,6]. While the female *D. medinensis* can emerge from any part of the host’s body, the gravid female worm frequently emerges from the host’s lower limb and releases first-stage larvae (L1) if the host comes into contact with water [4,8,10]. Infection with *D. medinensis* is rarely fatal, but worm emergence is accompanied by pain and incapacity, often lasting several weeks and causing devastating economic impacts in humans [10,12].

In 1986, The Carter Center (TCC) partnered with local Ministries of Health in GWD endemic countries to lead eradication efforts with support from the World Health Organization (WHO), the U.S. Centers for Disease Control and Prevention, and UNICEF [13,14]. Since this time, the annual incidence of human GWD cases has been lowered by over 99.9%, from an estimated 3.5 million cases reported in 1986 in 21 African and Asian countries [14] to 15 cases reported during 2024 in Chad and South Sudan [15]. The reduction in human GWD cases was achieved without the use of a pharmacological treatment by focusing instead on public health interventions aimed at interrupting the life cycle of *D. medinensis* such as increasing access to safe drinking water, treating bodies of water with temephos (an organophosphate that kills the intermediate host), educating communities on the life cycle of *D. medinensis* and signs of infection, increased disease surveillance, and a focus on containment of human cases and non-human animal infections [16,17]*.* Humans were believed to be the most common definitive host of *D. medinensis*, but infections were reported emerging from dogs in Chad in 2012 [18,19]. Since then, dog infections increased, peaking in 2019 when 1,934 dogs were reported as having an emerging *D. medinensis* worm before decreasing [20]. Despite significant progress toward eradication of *D. medinensis*, non-human animal infections, predominantly in domestic dogs and cats, impede eradication of this pathogen. Additionally, the role of wildlife in maintaining endemicity is suspected, but it is uncertain whether wildlife infections are primarily spillover from human cases or resulting from sylvatic transmission [21].

In 2024, Angola, Cameroon, Chad, Ethiopia, Mali, and South Sudan collectively reported 664 animal infections [15]. The *D. medinensis* worms that infect humans are genetically identical to those that infect dogs, cats, and wildlife [22,23], and the high number of *D. medinensis* infections in dogs is thought to drive transmission in most endemic countries [24]. An alternative mode of transmission, namely through the consumption of a transport (fish) or paratenic (frog, fish) host harboring copepods with L3s or L3s, respectively, may be contributing to the high disease burden in animals in Chad [25–29]. The Chadian National Guinea Worm Eradication Program (identified by its French acronym, PNEVG-T) began giving monetary incentives to owners who tethered their dogs with a suspected emerging *D. medinensis* to prevent them from contaminating water bodies [30]. Then, beginning in March 2020, the PNEVG-T encouraged villages with an annual incidence of five or more GW cases and infections to proactively tether their dogs [14]. Since the implementation of these interventions, *D. medinensis* infections in dogs decreased, but dog infections continue to outnumber human cases.

Flubendazole (FLBZ), a broad spectrum, benzimidazole anthelmintic drug, was proposed as a possible treatment for *D. medinensis* given its effectiveness as a macrofilaricide against medically relevant filarial nematode species in humans and animals [31]. While currently available in some European countries as an oral formulation for veterinary and human use to treat gastrointestinal nematode infections [31], a subcutaneous injection of FLBZ that creates a slowly dissolving drug depot had profound adulticidal activity against several nematode species and was proposed for treating systemic filarial infections [31,32]. FLBZ depot administration into the fatty tissue layer under the skin was inferred to allow for a slow and sustained release of the drug allowing for longer-lasting effects and reduced frequency of injections. L1s isolated from adult *D. medinensis* females recovered from FLBZ-treated ferrets (15 mg/kg administered parenterally 30 days apart) exhibited reduced motility resulting in <1% infection rate in copepods compared to 15% to 30% infection rate of larvae from worms from an untreated ferret [33]. FLBZ also damaged the uterine wall and caused degenerative changes of adult female *D. medinensis*, while adult females from the untreated ferret did not exhibit these degenerative changes [33].

A field trial (“FLBZ 1.0”) of owned Chadian dogs was conducted in which half of dogs in high-prevalence villages were randomized to receive one subcutaneous injection of FLBZ (15 mg/kg) or a control injection over three days and repeated three times every six months for 12 months [33]. This field trial found no significant difference in the number of emerging *D. medinensis* worms between FLBZ-treated and placebo-injected control dogs [33]. Limitations of this study included high attrition that could have reduced statistical power, and a 12-month follow-up period may not have been sufficient to observe possible effects of FLBZ that may have occurred past the first generation (first 12 months) of emerging worms. A study that examined possible effects of FLBZ beyond the one-year analysis using village-level surveillance data found a significant decrease in the predicted count of emerging GW infections in dogs residing in villages assigned to receive FLBZ compared to dogs residing in villages assigned to control approximately two years after the start of treatment in the context of other interventions [34]. In response to these challenges, a second field study (“FLBZ 2.0”) was executed in which randomization occurred at the village level to identify population-level effects alongside individual-level effects [35]. FLBZ 2.0 also had three separate recruitment rounds to combat study attrition, and the follow-up period lasted 18 months to identify possible longer-term effects of FLBZ.

The main aim of this current FLBZ 2.0 study was to identify the annual effect of FLBZ on emerging *D. medinensis* female worms over the study period compared to pre-treatment periods as illustrated in Fig 1. To better understand the mechanisms by which FLBZ impacts *D. medinensis*, we had the following sub-aims: 1) explore the population-level effects of FLBZ in the presence of other interventions; 2) evaluate the effects of FLBZ on the first generation of emerging *D. medinensis* and subcutaneous worms that could be *D. medinensis* female specimens in individual dogs; and 3) investigate the effect of FLBZ when administered subcutaneously by comparing the histopathology of adult female worms extracted from dogs treated with FLBZ and from control dogs. By analyzing these data annually, monthly, and at the individual dog and worm levels, we provide a more complete picture of the individual- and population-level effects of FLBZ on *D. medinensis.*

**Fig 1:**
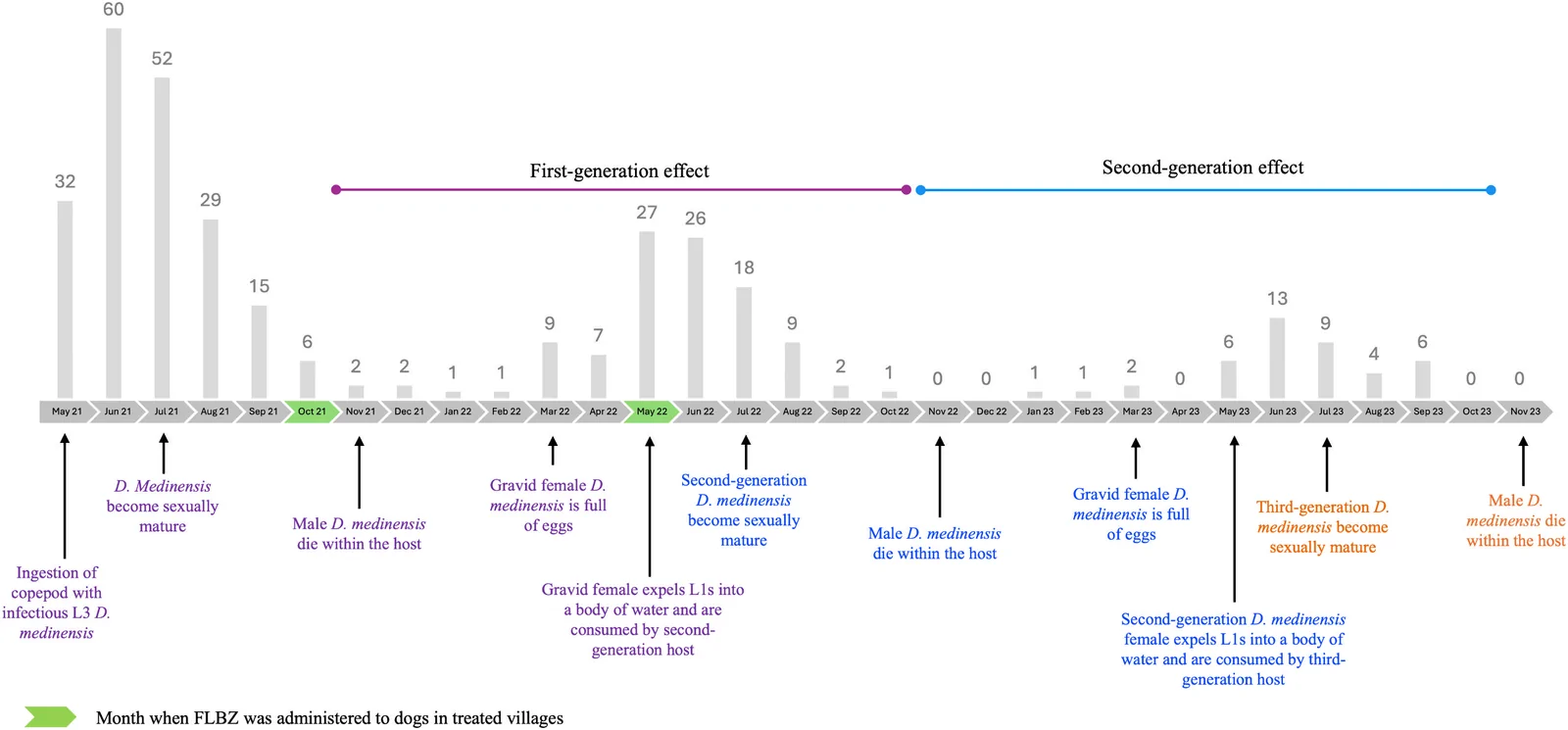
Timeline of *Dracunculus medinensis* life cycle and hypothesized course of action of flubendazole (FLBZ).

The life cycle illustrated represents the earliest timeline when we would likely observe an effect. Copepods are ingested at different times, which would shift the timeline at the top. A histogram shows with the total number of emerging female *D. medinensis* recorded from active surveillance from all enrolled villages.

## Materials and methods

### Ethics statement

The FLBZ 2.0 study received approval from the National Bioethics Committee of the Chadian Ministry of Higher Education, Research and Innovation (005/PR/MESRI/SE/DGM/CNBT/SG/2022) as well as the Institutional Animal Care and Use Committee of the University of Georgia (A2019 04 005). Dog owners were informed about study activities and provided verbal and written consent upon enrollment. Assent was confirmed at each follow-up round.

### Study area and population

Data for this study came from a cluster-randomized controlled trial that included villages along the western side of the Chari River randomly selected from the N’Djamena, Mayo-Kebbi Est, and Chari-Baguirmi regions in Chad (Fig 2). To be eligible for the study, villages must have been under active surveillance and be enrolled in the proactive tethering program at the start of the study. The policy for other interventions, including treatment of water bodies with temephos, was the same for all villages enrolled in the study. The method for selecting villages is described in Dupper et al. [35]. The final sample included 29 study villages randomized to treatment with FLBZ, and 27 study villages randomized to no treatment. Individual dogs located in study villages were enrolled if they were deemed to be in general good health, at least one year old, and were not pregnant or lactating at the time of enrollment. Enrollment of dogs eligible for inclusion occurred in October 2021, May 2022, and November 2022. Dogs enrolled in villages assigned to receive FLBZ were administered a subcutaneous injection of FLBZ (100 mg/kg, formulation described below) in October 2021 and May 2022. A third dose of FLBZ in November 2022 was planned for all active and newly enrolled dogs in villages assigned to treatment; however, logistical complications prevented this dose from being administered. All dogs were microchipped (Avid2028 9-digit chips, 125 kHz, Avid Identification Systems, Norco, CA) at enrollment to allow temporal tracking. Dog demographics including sex, weight, age, and history of GW infection were recorded at each enrollment period. The welfare of tethered dogs was continuously monitored by PNEVG-T veterinarians, and rabies vaccines were given to all dogs at the time of enrollment regardless of treatment status.

**Fig 2:**
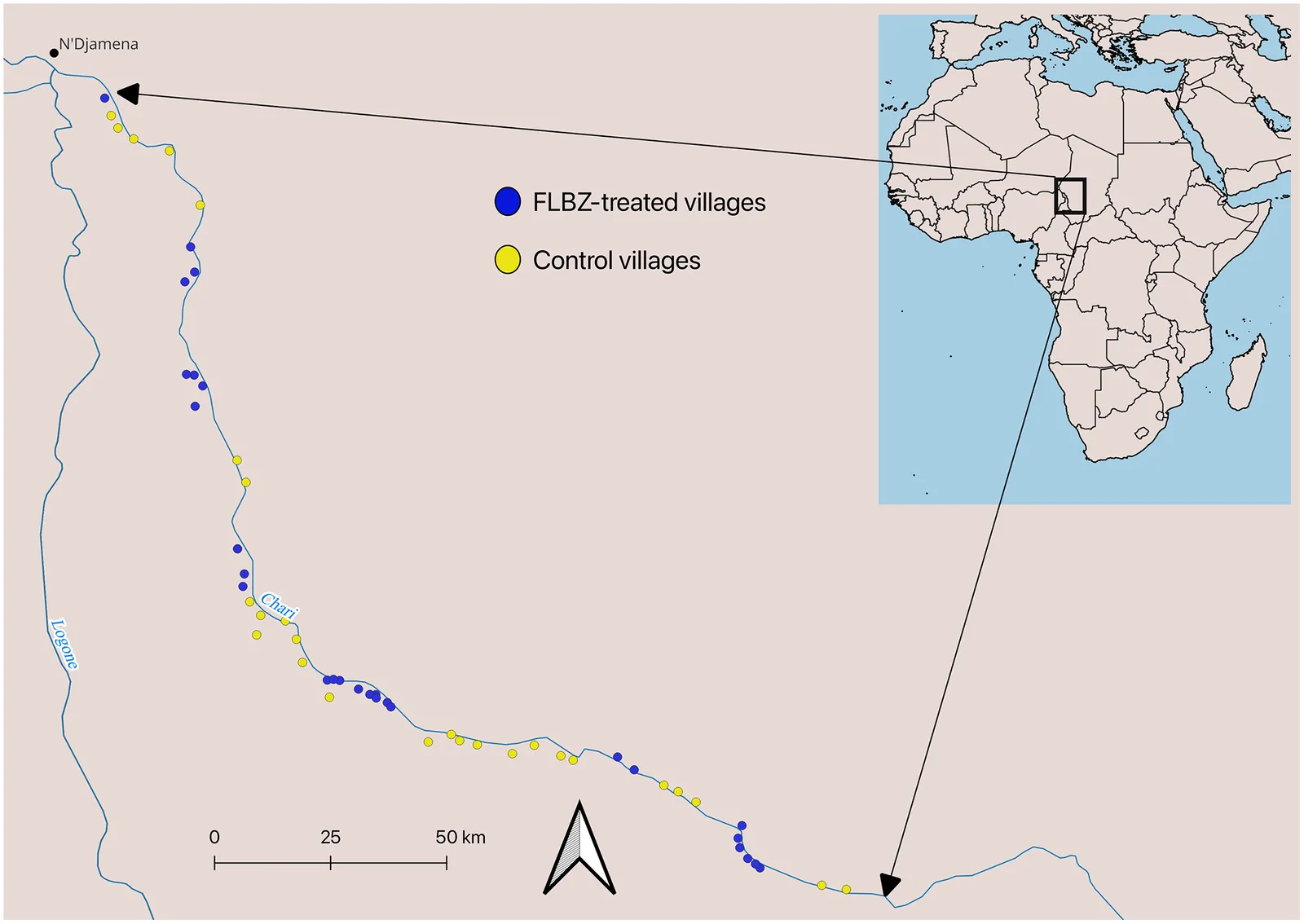
Map of villages randomized to receive flubendazole (treatment) or study activities but no treatment with flubendazole (control). The blue line represents the Chari River. All villages are clustered along the west side of the Chari River. Made with Natural Earth. Free vector and raster map data from naturalearthdata.com.

### Preparation of flubendazole (FLBZ) formulation

FLBZ was acquired from Janssen Pharmaceutical (Beerse, Belgium). All other materials were purchased from Merck KGaA (Darmstadt, Germany). A sterile aqueous formulation of FLBZ (500 mg/ml) was prepared using FLBZ, United State Pharmacopeia (USP)-grade purified water, 0.25% w/v hydroxyethylcellulose (HEC) and 0.1% w/v Tween 80. The sterile preparation of FLBZ formulation was conducted in a certified laminar flow hood and followed the guidelines as specified in USP < 797 > chapter for compounding sterile medications [36] and the Current Good Manufacturing Practices (cGMP) for pharmaceuticals [37]. The diluent was prepared using 0.25% w/v hydroxyethylcellulose and 0.1% v/v Tween 80 in water and was sterilized using a standard autoclaving protocol for liquids. The sterile diluent was used to prepare the FLBZ formulation using an Unguator electronic mortar (PCCA, Houston, TX). The FLBZ formulation was then aseptically placed into 50 ml sterile vials and labeled accordingly. Final FLBZ formulations were maintained under refrigeration (3–5°C) until up to one hour before administration in the field.

### Data

Outcome data for the annual, monthly, and individual analyses came from PNEVG-T active surveillance, through which village volunteers and supervisors visit all households in enrolled villages every 3–4 days and record cases of emerging *D. medinensis* in humans, domestic dogs, and domestic cats. We aggregated all reports of emerging *D. medinensis* in dogs in study villages by year or month for the different analyses. For the individual dog analysis, we matched outcome data from PNEVG-T to individual dogs in the FLBZ 2.0 study by owner and dog name. The Chad team was notified of names from enrolled villages that did not match dogs enrolled in the study, and a member of the Chad team visited households to resolve discrepancies.

#### Histopathology of *D. medinensis.*

To better determine whether FLBZ affects the reproductive morphology of gravid female *D. medinensis*, we compared the histological reproductive scores of gravid *D. medinensis* worms from treated and control dogs. All emerging *D. medinensis* worms from treated and control dogs enrolled in FLBZ 2.0 were collected and sent to Vassar College for genetic sequencing. Cuttings of worms extracted from FLBZ 2.0-enrolled villages were sent to Michigan State University Histopathology laboratory for analysis.

Using a modification of the method described in O’Neill et al., [38] the uteri of the worms were examined and scored based on the number of larvae present in that section: > 20 L1s present (A1), between 3 and 20 L1s present (A2), and <3 L1s present (A3). Other scoring designations included morulae present and mostly normal structures (M1), damaged morulae present (M2), an empty uterus but otherwise normal body structure (D1), and extensively damaged worm (D2). Worms that were too damaged to be classified or those from which only the anterior extremity was present were classified as unreadable (UR) (Fig 3).

**Fig 3:**
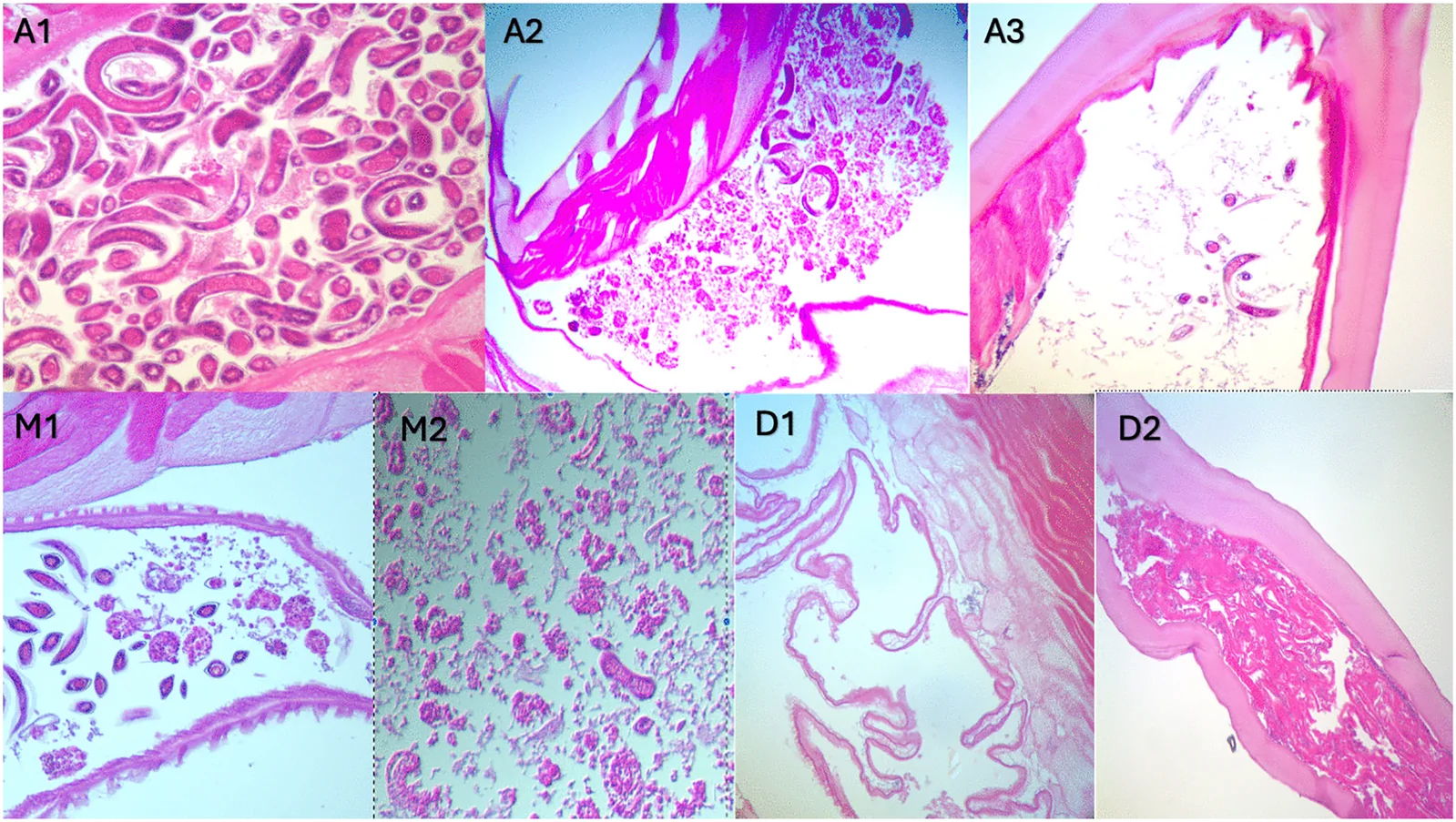
Graphics of scoring system of the reproductive morphology of gravid female *D. medinensis* from FLBZ treated and control dogs. Scoring based on the number of L1s present in the sample: (A1) >20 L1s present; (A2) 3–20 L1s present; and (A3) <3 L1s present. Other scoring designations included (M1) morulae present with normal structures; (M2) damaged morulae; (D1) empty uterus but normal body structure; and (D2) extensively damaged worm which is (UR) unreadable.

### Statistical analyses

#### Annual causal population effect of FLBZ.

All analyses and data visualizations were done in R (version 4.4.1). The main outcome of interest was the annual number of emerging *D. medinensis* in dogs at the village level beginning November 2019 and ending October 2023. To ensure comparable year-to-year data, we included data from November 2019 through October 2023, where each year began in November and ended in October. We chose to analyze these data using a difference-in-differences (DID) method. The DID method estimates the causal effect of treatment on the treated groups by comparing the average changes in outcomes between treatment and control groups in the population [39–41].

To perform our DID analysis, we included the following variables in the model: fixed effects for year (categorical), treatment status in FLBZ 1.0 (treated vs non-treated control), treatment status in FLBZ 2.0 (treated vs non-treated control), two DID estimands, and a random effect for village. The first DID estimand (first-generation effect) was an interaction term between time (first year post-treatment) and treatment village to estimate the average effect of FLBZ on the annual count of emerged *D. medinensis* in dogs from villages assigned to treatment in the first-year post-treatment. The second DID estimand (second-generation effect) was an interaction between time (second year post-treatment) and treatment village to estimate the average effect of FLBZ on the annual count of *D. medinensis* in dogs residing in villages assigned to treatment in the second year post-treatment. We initially fit a Poisson generalized linear mixed model (GLMM), but the variance was greater than the mean, so we then fit this model as a negative binomial GLMM. A likelihood ratio test showed that a negative binomial GLMM better fit the data. We then predicted the model fixed effects using the ‘predict’ function, and 95% confidence intervals were calculated through 1000 iterations of bootstrap samples using the ‘bootMer’ function, both available in the *lme4* package [42]. Covariates that had a p-value < 0.05 were considered statistically significant.

#### Monthly causal population effect of FLBZ.

As with the annual analysis, we conducted a DID analysis examining the average monthly change in emerging *D. medinensis* female worms in dogs due to FLBZ. The main outcome of interest for this portion of the study was the monthly count of emerging *D. medinensis* in dogs in study villages, aggregated at the village level. The study period began in January 2019 and extended through October 2023. Since the analysis evaluated outcomes on a monthly basis, it was necessary to account for time-variant seasonal differences. The final model included model-averaged fixed effects that best fit the effects of FLBZ from the 2019 study (FLBZ 1.0) and proactive tethering [34]. The variables for FLBZ included in the previous model reflected a continuous effect of FLBZ for six months after each injection, a second-generation effect of FLBZ beginning 12 months after the first injection, and an interaction between the second-generation effect of FLBZ and time. We found that a negative binomial GLMM provided the best fit for the data.

#### Individual dog analysis.

The individual dog analysis sought to more closely examine the first-generation effect of FLBZ in emerging *D. medinensis* worms and subcutaneous worms that could be *D. medinensis* in dogs that were treated with FLBZ. While emerging worms were recorded by the PNEVG-T surveillance team, the FLBZ 2.0 study teams recorded any subcutaneous worms that had not yet emerged but could be palpated during the physical examination conducted approximately every three months. Separate analyses were performed for emerged *D. medinensis* and for palpable subcutaneous worms that could be *D. medinensis* that had not emerged measuring whether a dog had at least one outcome detected over a 12-month period after enrollment. Due to FLBZ shipping delays, currently enrolled and newly enrolled dogs were not administered a dose of FLBZ in November 2022. Therefore, we restricted this analysis to include only those dogs enrolled in October 2021 and May 2022. Additionally, there were 17 dogs (26 total GW infections) that appeared in the surveillance data but were not matched to FLBZ 2.0-enrolled dogs, so these dogs were excluded from the analysis.

The inclusion of covariates for all analyses was determined using a directed acyclic graph (DAG) (S1 Fig). We first restricted the analysis to only dogs enrolled in October 2021 with the outcome a binary indicator for whether the dog had experienced at least one of the outcomes, respectively, within 12 months. We assessed the odds of experiencing an outcome in treated dogs compared to nontreated control dogs using an unadjusted and adjusted binomial GLMM including dog days of follow-up (centered and scaled) and a random effect for village. Sex at enrollment was initially included in the adjusted analysis, but removing this variable from the analysis did not change the effect estimate; therefore, it was removed from the adjusted analysis. We then assessed the odds of having an emerged *D. medinensis* worm or subcutaneous worm that could be *D. medinensis*, respectively, in treated compared to nontreated control dogs between June 2022 and May 2023, including all dogs enrolled in May 2022 and all dogs enrolled in October 2021 who were still alive in May 2022. As before, we ran an unadjusted and adjusted binomial GLMM controlled for dog days of follow-up (centered and scaled), an indicator variable for receipt of two FLBZ doses (yes/no), enrollment round (October 2021/May 2022), and a random effect for village.

#### Histopathology of *D. medinensis* in treated and nontreated control dogs.

We matched morphology scores to individual dogs in the study by village, dog and owner name, and date of worm emergence. The data were analyzed using a binomial GLMM with a random effect for dogs, given these dogs often had more than one emerging worm, and a fixed effect for any prior treatment with FLBZ in the FLBZ 2.0 trial. We collapsed morphology scores A1 and A2 together as normal reproductive morphology and potentially infectious (presence of apparently normal L1) and all other scores as non-normal reproductive morphology with little potential infectiousness after consulting with the Guinea Worm Therapeutics Working Group prior to observing the results. The one worm that was unreadable (UR) was removed from the main analysis. We also ran a sensitivity analysis to examine whether different groupings of our outcome variable (histological morphology score) would result in different estimates of effect. We performed a per-protocol analysis, so the main exposure of interest was receipt of FLBZ (yes vs no) at time of worm emergence, instead of FLBZ treatment assignment. Dogs with an emerging worm before treatment with FLBZ were considered not treated even if they were treated after worm emergence.

We identified possible confounders through a DAG (S2 Fig). Adjusting for receipt of two FLBZ injections over the study period closed all biasing pathways between treatment with FLBZ and reproductive morphology score. Female *D. medinensis* specimens for which a reproductive morphology score could not be determined were removed from the initial round of analysis. Lastly, we performed a sensitivity analysis assuming that all worms with no morphology score had normal reproductive morphology, or all did not have normal reproductive morphology, to determine if our conclusions would change based on these assumptions (S1 Table).

## Results

### Demographics of dogs

The methods for randomizing village clusters to FLBZ or to control have been described previously [35] and are presented in Fig 4. A total of 56 villages were enrolled in the study, 29 were randomized to the FLBZ arm, and 27 were randomized to the control arm. There was no significant difference in the number of male and female dogs enrolled in each study arm, and the median age was similar for both study arms, confirming that the demographics of the enrolled dogs were similar at baseline (Table 1). The weight (in kg) of dogs at enrollment was slightly higher in the control arm compared to dogs in the treatment arm on a Student’s t-test, however, there was no difference in enrollment weight when comparing dogs in the treated and control arms by enrollment rounds (see Individual dog analysis section).

**Table 1:** Baseline demographic information for each study arm given at the village and individual dog level.

	FLBZ group	Control group
**Village factors at baseline**		
Number	29	27
Median dog population (IQR)	28 (22 – 65)	42 (18 – 56)
**Dog factors at baseline**		
Number	1424	1079
Median age in years (IQR)	2 (1 – 3)	2 (1 – 3)
Sex (%)		
Male	787 (55.3)	576 (53.4)
Female	637 (44.7)	503 (46.6)
Median weight in kg (IQR)^*^	14 (12 – 16)	14 (12 – 17)

Abbreviations: FLBZ, flubendazole; IQR, interquartile range

* p < 0.05

**Fig 4:**
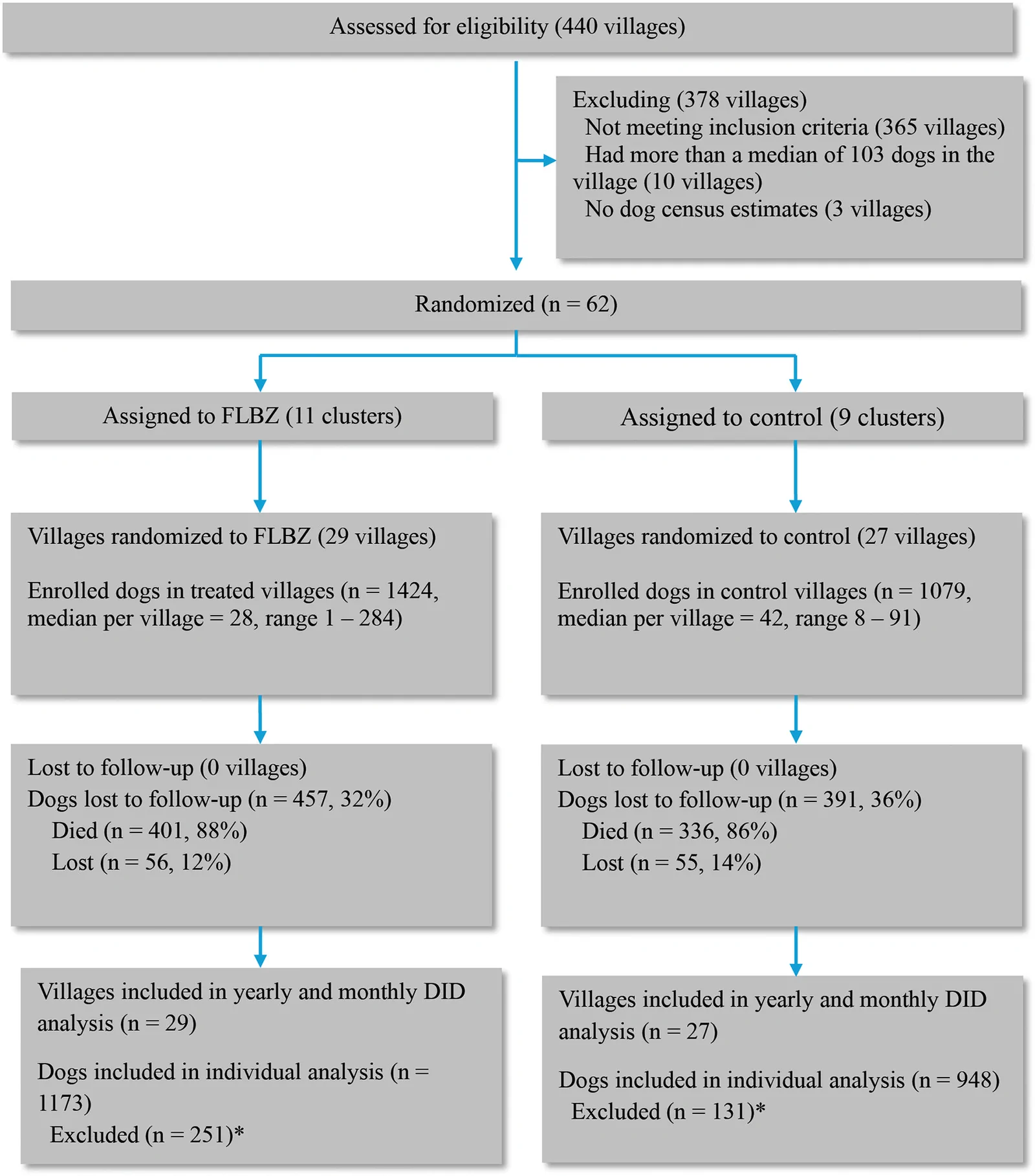
Flow diagram of selection of village clusters and individual dogs through phases of clinical trial. ^*^ Dogs enrolled in November 2022 were excluded from the individual dog analysis because these dogs did not receive a dose of FLBZ at enrollment.

### Annual causal population effect of FLBZ

To assess the effect of FLBZ on the first- and second-generation emerging *D. medinensis* worms in dogs residing in villages assigned to treatment compared with those in villages assigned to control, we used a negative binomial GLMM with two DID coefficients. While the count of emerging *D. medinensis* declined each year, we did not observe a significant reduction in the expected count of emerging *D. medinensis* in the first year of the study compared to pre-treatment counts (Table 2). However, there was a significant reduction in the expected ratio of the number of emerging *D. medinensis* worms in dogs in the second year compared to post-treatment counts (p = 0.03). We then estimated the predicted number of emerging *D. medinensis* worms in dogs comparing treated and control villages with the 95% confidence intervals in the pre- and post-treatment period (Fig 5). Based on these predictions, in the final year of the study, treated villages would have 59.3% fewer emerging *D. medinensis* worms per village per year (95% CI: 53.8% fewer to 69.2% fewer infections per village per year) in dogs compared to control villages. The yearly downward trend in the ratio of counts of emerging *D. medinensis* worms independent of treatment can be attributed to concurrent interventions to reduce GW incidence that were currently active at the time of this study.

**Table 2:** Model fixed effects for the annual average ratio of counts of emerging *Dracunculus medinensis.*

Covariate	Ratio of Counts Estimate	p-value
Treated in FLBZ 1.0	1.25	0.45
Treatment arm	0.94	0.80
Year		
Nov 19 – Oct 20	Ref	
Nov 20 – Oct 21	0.32	<0.001
Nov 21 – Oct 22	0.14	<0.001
Nov 22 – Oct 23	0.09	<0.001
First-generation effect of treatment^*^	1.01	0.98
Second-generation effect of treatment^†^	0.43	0.03

* The DID estimate for the average effect of treatment on the dogs residing in villages assigned to treatment between November 2021 and October 2022.

† The DID estimate for the average effect of treatment on the dogs residing in villages assigned to treatment between November 2022 and October 2023.

**Fig 5:**
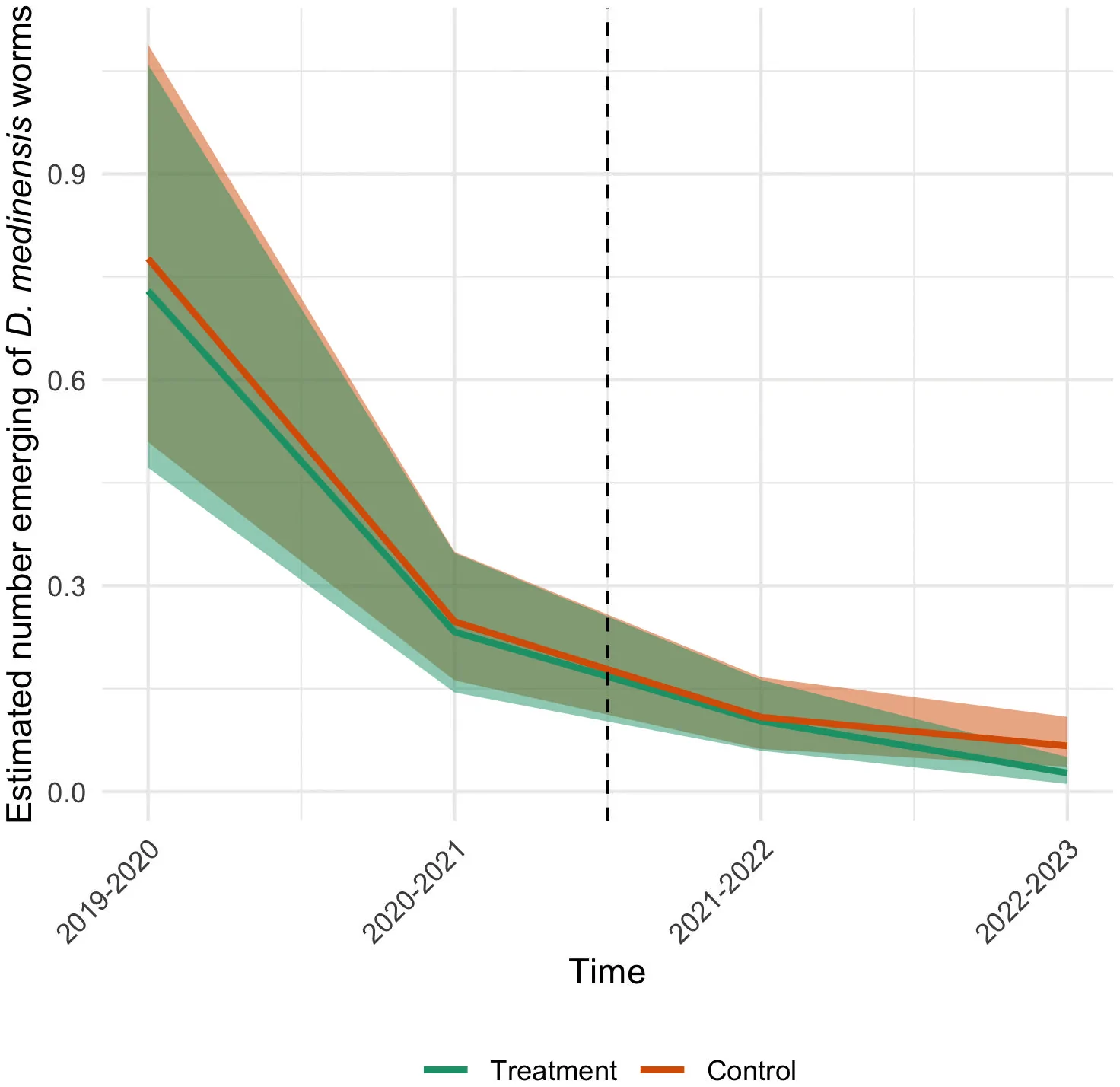
Estimated number of emerging *Dracunculus medinensis* worms in dogs per village between November 2019 and October 2023. The dotted line represents the start of the FLBZ 2.0 study in October 2021, and the shaded areas show the 95% confidence intervals.

### Monthly causal effect of FLBZ

As with the annual analysis, we fit a negative binomial GLMM with separate DID coefficients estimating the first- and second-generation effects of FLBZ on the percentage change in *D. medinensis* emergence in dogs from treated villages averaged over a year while holding the effects of past and concurrent interventions constant. The effects of the first-generation FLBZ 2.0 treatment in dogs residing in villages assigned to treatment were not statistically significant (Table 3). However, the second-year post-treatment with FLBZ resulted in a statistically significant 94% decrease (95% CI: 39% decrease to 99% decrease; p = 0.02) in emerging *D. medinensis* worms in dogs from treated villages, from pre-treatment to post-treatment after controlling for time-variant fixed effects.

**Table 3:** Model fixed effects for the monthly average ratio of counts of emerging *Dracunculus medinensis* in dogs per village.

Covariate	Ratio of Counts Estimate	p-value
Month of Study Period	0.98	0.01
Season	2.41	<0.01
Treated in FLBZ 1.0	1.67	0.15
1^st^ generation continuous effect of FLBZ 1.0	1.42	0.13
2^nd^ generation continuous effect of FLBZ 1.0	0.95	0.83
Time since 2^nd^-generation effect of FLBZ 1.0 in treated villages	0.98	0.18
1^st^ generation effect of proactive tethering	0.80	0.17
2^nd^ generation effect of proactive tethering	0.52	<0.01
Time since 2^nd^ generation effect of proactive tethering	1.00	0.89
FLBZ 2.0 treatment start	0.52	0.03
Time since FLBZ 2.0 treatment start	1.01	0.77
FLBZ 2.0 treatment arm	1.03	0.92
DID estimate for 1^st^ generation continuous effect of FLBZ 2.0	1.22	0.47
DID estimate for 2^nd^ generation effect of FLBZ 2.0	0.06	0.02
Time since FLBZ 2.0 treatment start	1.25	0.09

We ran predictions using the monthly negative binomial GLMM to further tease out the population-level effects of the two FLBZ trials on the number of emerging *D. medinensis* worms from dogs who were 1) not treated in FLBZ 1.0 or FLBZ 2.0, 2) treated in FLBZ 1.0 but not treated in FLBZ 2.0, 3) not treated in FLBZ 1.0 but treated in FLBZ 2.0, and 4) treated in FLBZ 1.0 and FLBZ 2.0. We then fit the predictions for each scenario to the raw data to check model fit (Fig 6). Villages with high worm burdens were intentionally chosen for inclusion in FLBZ 1.0, which can explain the high numbers of emerging *D. medinensis* worms in FLBZ 1.0 treated villages seen in Fig 6. When comparing the average number of emerging *D. medinensis* worms in dogs during the final year of the study (November 2022 – October 2023), holding all other variables constant, dogs not treated in FLBZ 1.0 or FLBZ 2.0 had the highest number of emerging *D. medinensis* (82 worms per 100 villages per year, 95% CI: 28 worms to 244 worms per 100 villages per year), followed by dogs treated in FLBZ 1.0 but not treated in FLBZ 2.0 (70 worms per 100 villages per year, 95% CI: 23 worms to 167 worms per 100 villages per year), dogs not treated in FLBZ 1.0 but treated in FLBZ 2.0 (34 worms per 100 villages per year, 95% CI: 10 worms to 66 worms per 100 villages per year), and treated in FLBZ 1.0 and FLBZ 2.0 (24 worms per 100 villages per year, 95% CI: 6 worms to 54 worms per 100 villages per year) (Fig 7).

**Fig 6:**
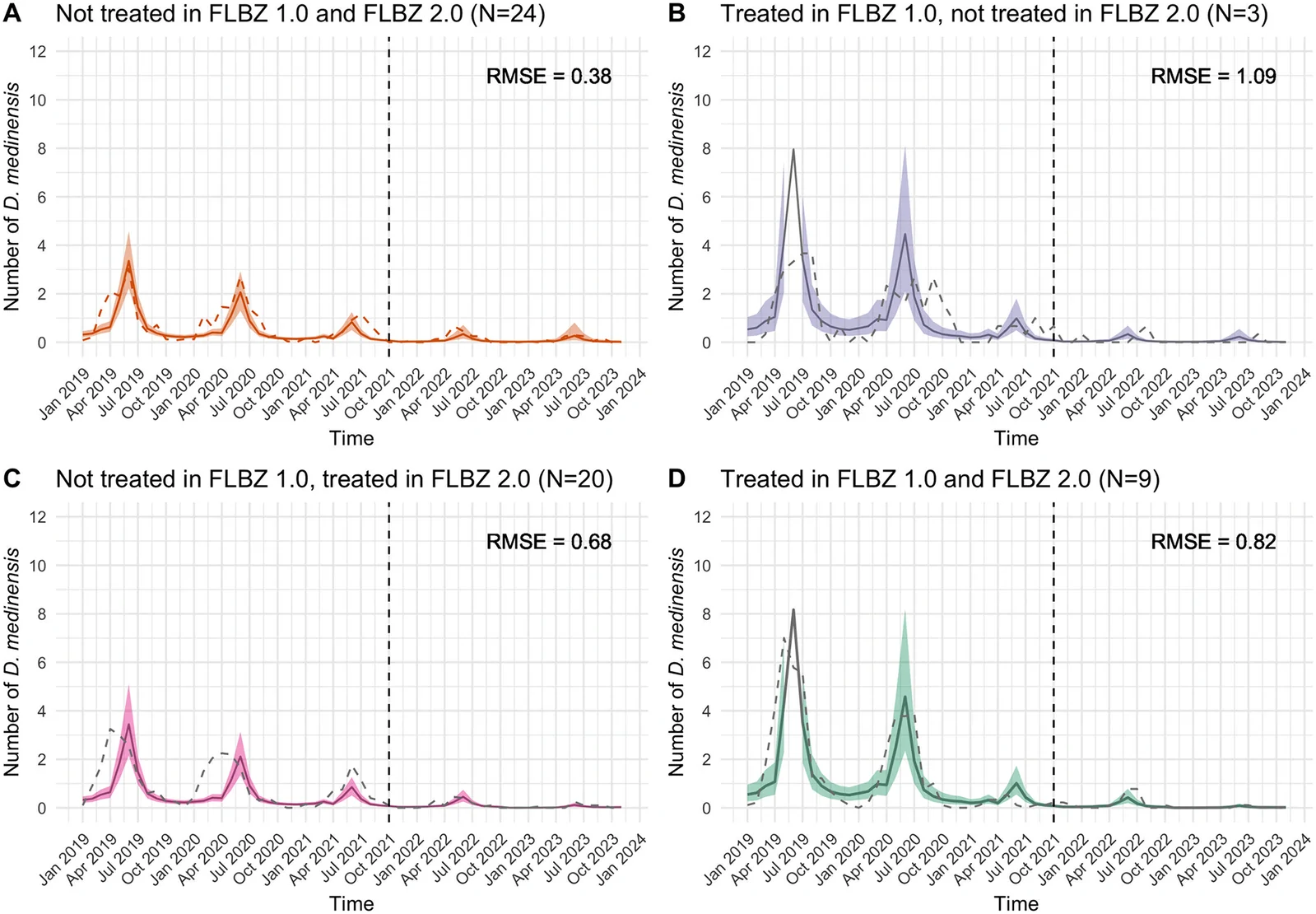
Estimated count of emerging *Dracunculus medinensis* from the monthly negative binomial generalized linear mixed model overlayed with the raw number of emerging *D. medinensis* worms in the 56 enrolled FLBZ 2.0 study villages from January 2019 through November 2023. The vertical dotted line indicates the start of FLBZ 2.0. The Root Mean Square Error (RMSE) is aggregated over each month. Lower RMSE values indicate better agreement between observed and predicted worm counts. The vertical dotted line indicates the start of FLBZ 2.0, and the shaded areas show the 95% confidence intervals.

**Fig 7:**
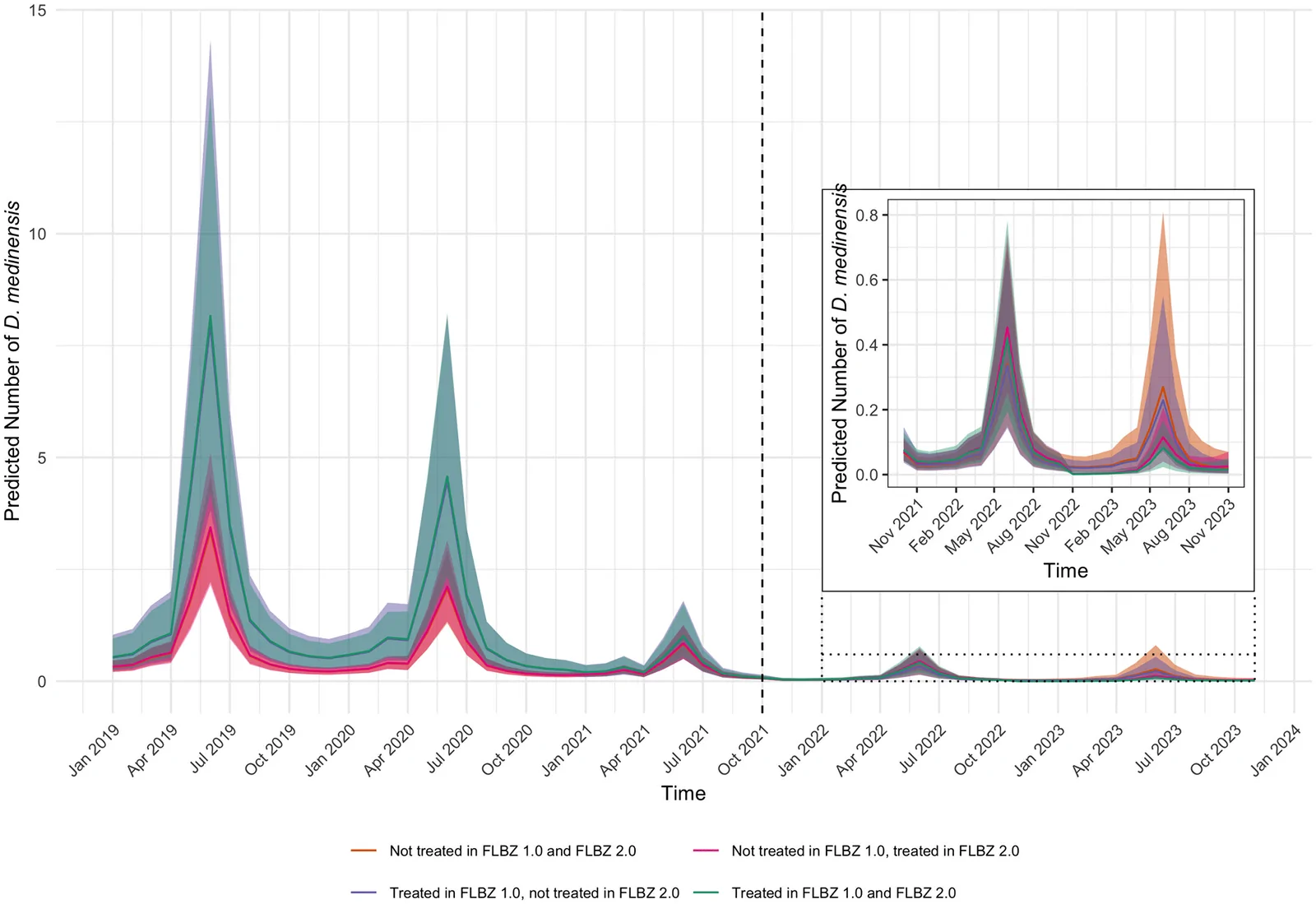
Estimated count of emerging *Dracunculus medinensis* worms in dogs based on fixed effects of negative binomial generalized linear mixed model from January 2019 through November 2023.

### Individual dog analysis

We first examined the demographic characteristics of the individual dogs enrolled in the study by enrollment round and treatment status (Table 4). Only in the first enrollment round in October 2021 were there statistically significant differences in sex at enrollment (p < 0.05). We detected no statistically significant differences in enrollment age or weight in either enrollment round, thus confirming that the randomization was successful.

**Table 4:** Demographics of individual dogs enrolled in FLBZ 2.0 stratified by enrollment period.

	Enrolled in October 2021 (N = 1,204)				Enrolled in May 2022 (N = 917)			
	FLBZ		Control		FLBZ		Control	
	N	%	N	%	N	%	N	%
Sex^*^								
Male	446	69.0	346	62.0	215	40.8	164	42.1
Female	200	31.0	212	38.0	312	59.2	226	57.9
Age (years) (median, IQR)	2.0	1.0, 4.0	2.0	1.0, 3.0	2.0	1.0, 3.0	2.0	1.0, 3.0
Weight in kg (median, IQR)	14.5	12.4, 16.8	14.9	12.4, 17.3	14.0	12.0, 16.0	14.0	11.0, 16.0
Dogs having at least one emerging GW over the 12-month period								
Yes	15	2.3	15	2.7	9	1.7	9	2.3
No	631	97.7	543	97.3	518	98.3	381	97.7
Dogs having at least one subcutaneous worm over the 12-month period								
Yes	57	8.8	29	5.2	29	5.5	15	3.8
No	589	91.2	529	94.8	498	94.5	375	96.2
Follow-up Time (days) (median, IQR)	362	203, 369	357	195, 368	366	193, 369	365	0, 367

Abbreviations: IQR, interquartile range; FLBZ, flubendazole; GW, Guinea worm

* p < 0.05

There was no statistically significant difference in the odds of having an emerging *D. medinensis* worm within 12 months after enrollment between dogs treated with FLBZ and untreated dogs in either analysis (Table 5). Among dogs enrolled in October 2021, dogs assigned to receive treatment had 2.4 higher odds of having at least one subcutaneous worm that could be *D. medinensis* within 12 months after enrollment compared to dogs assigned to no treatment in unadjusted (odds ratio [OR] = 2.4, 95% CI: 1.1 – 5.2) and adjusted analyses (OR = 2.4, 95% CI: 1.1 – 5.4) (Table 6). However, with the inclusion of dogs enrolled in May 2022, this association was no longer statistically significant.

**Table 5:** The unadjusted and adjusted odds of having at least one emerging worm at each follow-up period comparing individual dogs treated with FLBZ to individual dogs receiving no treatment.

October 2021				October 2021 & May 2022			
Unadjusted OR		Adjusted OR*		Unadjusted OR		Adjusted OR**	
OR	95% CI	OR	95% CI	OR	95% CI	OR	95% CI
1.0	0.3, 2.6	1.0	0.3, 2.8	0.7	0.3, 1.6	0.8	0.3, 2.5

Abbreviations: OR, odds ratio; CI, confidence intervals; FLBZ, flubendazole*Adjusted for dog days at risk (centered and scaled).**Adjusted for dog days at risk (centered and scaled), number of FLBZ doses, and enrollment round.

**Table 6:** The unadjusted and adjusted odds of having at least one subcutaneous worm at each follow-up period comparing individual dogs treated with FLBZ to individual dogs receiving no treatment.

October 2021				October 2021 & May 2022			
Unadjusted OR		Adjusted OR*		Unadjusted OR		Adjusted OR**	
OR	95% CI	OR	95% CI	OR	95% CI	OR	95% CI
2.4	1.1, 5.2	2.4	1.1, 5.4	1.5	0.6, 3.6	2.4	0.8, 6.7

Abbreviations: OR, odds ratio; CI, confidence intervals; FLBZ, flubendazole*Adjusted for dog days at risk (centered and scaled).

**Adjusted for dog days at risk (centered and scaled), number of FLBZ doses, and enrollment round.

#### Dracunculus medinensis *histopathology.*

A total of 102 emerging female nematodes were recovered between October 2021 and May 2023 from treated (N = 32, 31%) and non-treated (N = 70, 69%) dogs enrolled in the FLBZ 2.0 trial (Table 7). In the negative binomial GLMM, a dog with at least one FLBZ injection had 80% lower odds (CI: 97.6% lower to 10.9% lower) of having an emerging *D. medinensis* worm with normal morphology (a morphology score of A1 or A2) compared to a dog that did not receive a FLBZ injection at or before the time of worm emergence, adjusting for number of FLBZ injections received and random effect for dog. We additionally performed a sensitivity analysis in which the unreadable worm was categorized as having normal morphology and then as not having normal morphology, but these analyses did not alter our conclusions (S1 Table).

**Table 7:** Number (%) of *Dracunculus medinensis* females that emerged from FLBZ-treated and non-treated dogs by histopathology scores.

Histopathology Scores				
	A1	A2	A3	Other^*^
FLBZ	6 (18.8)	1 (3.1)	6 (18.8)	19 (59.3)
Control	28 (40.0)	6 (8.6)	4 (5.7)	32 (45.7)

* Other includes empty uterus but relatively normal body structure (D1), worm damaged (D2), morulae present but mostly normal structures (M1), damaged morulae (M2), unreadable (UR)

When categorizing scores of A1 only or A1, A2, and A3 as normal reproductive morphology compared to all other scores, we continued to see lower odds of having an emerging *D. medinensis* worm with normal morphology after receiving at least one injection of FLBZ, compared to dogs that did not receive an injection of FLBZ, but neither of these categorizations reached statistical significance.

## Discussion

We sought to examine the first- and second-generation effects of FLBZ on emerging *D. medinensis* worms at the village, individual dog, and worm levels. This clinical trial attempted to address challenges encountered during FLBZ 1.0 including high attrition, short follow-up periods, and feasibility of drug administration. Regarding drug dose, during FLBZ 1.0, 15 mg/kg of FLBZ (0.375% hydroxyethylcellulose and 0.1% Tween 80) was administered to dogs for three consecutive days and repeated every six months, whereas 100 mg/kg of FLBZ (0.25% hydroxyethylcellulose and 0.1% Tween 80) was administered to dogs once every six months in FLBZ 2.0. FLBZ was administered to dogs in a formulation that was designed to form a depot at the injection site and to slowly release the drug into the dogs’ tissues over six months. This differed from previous studies that subcutaneously administered FLBZ ranging between 12.5 and 400 mg/kg (1% Tween 80 in water) in cats experimentally infected with 100 infective *Brugia pahangi* larvae [43] and administered 1, 3, or 5 subcutaneous injections of 50 mg/kg of FLBZ (20% FLBZ in sodium citrate solution) to puppies experimentally infected with 50 *Dirofilaria immitis* larvae [44]. These studies found that most adult worms were killed in cats receiving 100 mg/kg or more of FLBZ, and puppies receiving 5 FLBZ injections two months after larvae inoculation prevented worm development into the adult stages [43,44]. The addition of a depot allowing a slow release of FLBZ over time in FLBZ 1.0 and FLBZ 2.0 theoretically allowed the drug to remain active in the dogs’ tissues allowing for fewer follow-ups (Fig 1). One depot injection of FLBZ used in the current study provides logistical advantages over multiple repeated injections when working in challenging settings and could be deployed alongside existing interventions.

Regarding differentiation of effects of the study from effects of the drug, which could not be differentiated in our retrospective analysis of FLBZ 1.0 data [34], the difference can be observed in the monthly DID analysis where we predicted the effects of FLBZ 1.0 and FLBZ 2.0 separately and combined using village level data between January 2019 and November 2023. The lowest annual number of emerging *D. medinensis* worms per 100 villages was observed in villages that were treated in both FLBZ 1.0 and FLBZ 2.0. In FLBZ 1.0, half of the dogs in each village were treated with FLBZ at a lower dose, while all dogs in treated villages in FLBZ 2.0 received FLBZ, which likely also contributed to differences in effects between the two trials. This can be considered a population-level dose-response with those villages treated only in FLBZ 2.0 seeing a greater decrease in emerging worms compared with those only treated in FLBZ 1.0, and villages treated in both trials seeing the largest decrease. All analyzed villages were enrolled in the FLBZ 2.0 study, so the larger effect observed in FLBZ 2.0 suggests that the effect was due to the drug and not the other study activities, which were the same between the two arms in FLBZ 2.0. It should be noted that the FLBZ 2.0 study was powered for the primary, annual analysis, as described in Dupper et al. [35], so the lack of significance in the secondary, monthly analyses, though unfortunate, is expected.

We found no association between treatment with FLBZ and the odds of having an emerging *D. medinensis* worm compared to the non-treated control group over 12 months in unadjusted and adjusted analyses at the individual dog level. This finding suggests that 100 mg/kg of FLBZ in this formulation did not kill *D. medinensis* larvae and adults in treated dogs as different formulations did with *Brugia pahangi* and *Dirofilaria immitis* in experimental studies [43,44].

When examining subcutaneous worms that could be *D. medinensis* as the outcome, we found higher odds of having a subcutaneous worm in FLBZ-treated dogs compared to control dogs over 12 months of follow-up when including only dogs enrolled in October 2021. The finding suggested that FLBZ-treated dogs had higher odds of subcutaneous worms detected on physical examination, which is not a biologically meaningful association, thus we explored possible explanations. All dogs were given physical examinations by the study team at each follow-up, and probable subcutaneous worms that could be *D. medinensis* were recorded. Because study teams were not blinded to village treatment assignment, this significant finding could be a result of detection bias, wherein study teams assigned to treatment villages may have been more thorough in dog examinations than study teams assigned to control villages, thus increasing the odds of palpating a subcutaneous worm. Another explanation is that October 2021 was also the enrollment round in which there were significantly more male dogs in the FLBZ arm, so any behavior that differed between male and female dogs could contribute to this difference in subcutaneous worms. A study of domestic free-roaming dogs in Australia found increased roaming behavior and thus exposure to surface water in male dogs [45]. However, similar studies in Chad did not find a statistically significant association between roaming behavior and dog sex [28,46]. One study on the roaming behavior of dogs in Chad found that over 70% of dogs stayed within the village boundaries, but roaming increased during the dry season and was highly dependent on owner hunting activities [47].

While the number of suspected subcutaneous worms may have differed in this one round, it should be remembered that there was no difference in the odds of having an emerging *D. medinensis* worm between FLBZ-treated and control villages, and that not all subcutaneous worms emerge or are *D. medinensis*. Only 13% of gravid female *D. insignis,* a related parasitic nematode found in North America, found on necropsy of experimentally infected ferrets emerged, and over 90% of the non-emergent worms contained larvae that were not viable [48]. Therefore, worms that were palpated in FLBZ-treated dogs may not be triggered to emerge because the larvae in the gravid female worm are not fully developed or not viable. While this is not strong evidence, it further supports the hypothesis that FLBZ at the dose and formulation in FLBZ 2.0 impacts L1 development rather than development from L3 to adult and survival of adults.

Relatedly, we found that *D. medinensis* worms collected from FLBZ-treated dogs enrolled in FLBZ 2.0 had lower odds of having normal morphology compared to *D. medinensis* worms collected from FLBZ-enrolled control dogs. These results also support the findings from a previous study that found GW larvae recovered from ferrets and dogs treated with FLBZ exhibited inhibited motility and reduced viability to infect copepods compared to GW larvae recovered from a ferret and dogs not treated with FLBZ [33]. Extracted worms came from the PNEVG-T active surveillance program, so the method of collection should be the same regardless of study arm minimizing bias from collection method. That said, controlled immersion, which is often used to help facilitate the extraction of worms and L1 larvae from the emerging gravid female worms, may have influenced histopathological assessments of collected worms. Taken together, these analyses point to FLBZ affecting the development of L1s within the gravid female worm so that the larvae have limited viability to infect copepods and subsequently the second generation of mammalian hosts.

Our study has several limitations. While this was a randomized clinical trial, it was conducted under field conditions in which not all biasing variables were known or controllable. We attempted to mitigate this issue by enrolling villages with similar dog husbandry, GW intervention practices and surveillance intensity. Our outcome data came from surveillance data, which was collected by PNEVG-T separately from the field trial, thus mitigating bias in outcome data collection. While it was our goal to enroll all eligible dogs in each enrolled village, this did not always happen, not due to refusals, but due to the presence of some unowned dogs and dogs that the program was not aware of. Non-enrollment of eligible dogs could have attenuated the effects of FLBZ observed in the annual and monthly DID analyses. For our individual analysis, we matched surveillance data to our list of enrolled dogs using the names of the dog and dog owner through fuzzy matching. In several instances, the names of dogs in surveillance data did not match FLBZ 2.0-enrolled dogs. A member of the Chad team visited households of FLBZ 2.0-enrolled dogs to settle outstanding discrepancies in the data; however, 17 unreported dogs had 26 GW infections in enrolled villages even after this reconciliation. Lastly, and a major limitation of this study, a contemporaneous pharmacokinetic study in laboratory dogs in the USA using the same FLBZ formulation did not detect FLBZ exposure at the expected consistent levels in plasma from treated dogs; it instead found inconsistent spikes in levels in 13 dogs and no detection in three dogs [49]. Serum collected from dogs during FLBZ 2.0 was not collected with the intent of testing for FLBZ levels so was not in appropriate volumes or rapidly taken to a laboratory with this capability. Additionally, we did not collect subcutaneous tissues, so the actual exposure to FLBZ in treated dogs is unknown and limits how much causality can be inferred from this study. If a spike in FLBZ plasma occurred at the time a dog had a developing *D. medinensis* worm, then its viability could be affected and produce the observed effect. Another possibility is that the therapeutic effect we observed is due to detection bias because program staff and study teams were aware of treatment assignment. However, all analyses described in this paper consistently suggest that this regimen of FLBZ reduces the number of viable L1 in *D. medinensis* females emerging from treated dogs.

While we cannot be certain how much FLBZ was present in the tissues of dogs treated with FLBZ, the main annual DID analysis demonstrated that this regimen of FLBZ resulted in cluster-level coverage effects, which reduced the annual count of emerging *D. medinensis* in dogs from treated villages in the second-generation, or second seasonal cycle, compared to pre-trial counts. This result is supported by the monthly DID, individual dog analysis, and histological analysis. Instead of reducing emerging *D. medinensis* worms in treated dogs, FLBZ appears to inhibit embryogenesis of developing L1s within the gravid female worm making them less likely to infect copepods and thus interrupting the transmission cycle. Parenteral injections of FLBZ in a depot strategy may be an effective treatment to reduce emerging *D. medinensis* worms in domestic dogs in endemic communities in the context of other control efforts.

## Supporting information

S1 Fig Directed acyclic graph (DAG) of the relationship between treatment with flubendazole (FLBZ) and emerging *D. medinensis* worms/subcutaneous worms that could be *D. medinensis.* (DOCX)

S2 Fig Directed acyclic graph (DAG) of the relationship between host exposure to FLBZ and emerging *D. medinensis* female worm uterine score. (DOCX)

S1 Table Sensitivity analysis of upper and lower boundaries for estimates assuming all unreadable (UR) uterine worm scores did not have normal morphology or did have normal morphology. (DOCX)

S2 Table Minimal data set of village level and dog level data enrolled in the flubendazole cluster randomized controlled trial between October 2021 and June 2023. (XLSX)
