# Supplementary material for: Flubendazole reduces emerging Dracunculus medinensis in dogs in Chad: Results from a two-year study
Source: PLoS Negl Trop Dis. 2026 Sep 25;20(9):e0014763. doi: 10.1371/journal.pntd.0014763 (PMC13630316; doi:10.1371/journal.pntd.0014763)
Supplement: Supplementary file 1 [file pntd.0014763.s001.docx]

S1 Fig. Directed acyclic graph (DAG) of the relationship between treatment with flubendazole (FLBZ) and emerging/subcutaneous *D. medinensis* worms. Blue circles signify observed covariates, green circles signify independent exposure, orange circle signifies dependent outcomes, and grey circles signify unobserved covariables.
