# Supplementary material for: Flubendazole reduces emerging Dracunculus medinensis in dogs in Chad: Results from a two-year study
Source: PLoS Negl Trop Dis. 2026 Sep 25;20(9):e0014763. doi: 10.1371/journal.pntd.0014763 (PMC13630316; doi:10.1371/journal.pntd.0014763)
Supplement: Supplementary file 2 [file pntd.0014763.s002.docx]

S2 Fig. Directed acyclic graph (DAG) of the relationship between host exposure to FLBZ and emerging *D. medinensis* female worm uterine score. Blue circles signify observed covariates, green circles signify independent exposure, orange circle signifies dependent outcomes, and grey circles signify unobserved covariables.
