# Supplementary material for: Flubendazole reduces emerging Dracunculus medinensis in dogs in Chad: Results from a two-year study
Source: PLoS Negl Trop Dis. 2026 Sep 25;20(9):e0014763. doi: 10.1371/journal.pntd.0014763 (PMC13630316; doi:10.1371/journal.pntd.0014763)
Supplement: Supplementary file 3 [file pntd.0014763.s003.docx]

S1 Table. Sensitivity analysis of upper and lower boundaries for estimates assuming all unreadable (UR) uterine worm scores did not have normal morphology or did have normal morphology

| Worm uterine score | OR | 95% CI |
| --- | --- | --- |
| Assuming all “UR” have non-normal reproductive morphology | 0.3 | 0.1, 1.0 |
| Assuming all “UR” have normal reproductive morphology | 0.2 | 0.1, 0.9 |

Abbreviations: OR, odds ratio; CI, confidence interval
